# The Evolution of the *Anopheles* 16 Genomes Project

**DOI:** 10.1534/g3.113.006247

**Published:** 2013-07-01

**Authors:** Daniel E. Neafsey, George K. Christophides, Frank H. Collins, Scott J. Emrich, Michael C. Fontaine, William Gelbart, Matthew W. Hahn, Paul I. Howell, Fotis C. Kafatos, Daniel Lawson, Marc A. T. Muskavitch, Robert M. Waterhouse, Louise J. Williams, Nora J. Besansky

**Affiliations:** * Broad Institute, Cambridge, Massachusetts 02142; † Imperial College London, South Kensington Campus, London SW7 2AZ, United Kingdom; ‡ Eck Institute for Global Health, University of Notre Dame, Notre Dame, Indianapolis 46556; § Harvard University, Cambridge, Massachusetts 02138; ** Indiana University, Bloomington, Indiana 47405; †† Centers for Disease Control and Prevention, Atlanta, Georgia 30341; ‡‡ European Bioinformatics Institute, Wellcome Trust Genome Campus, Hinxton, Cambridge CB10 1SD United Kingdom; §§ Boston College, Chestnut Hill, Massachusetts 02467; *** Harvard School of Public Health, Boston, Massachusetts 02115; ††† Massachusetts Institute of Technology, Cambridge, Massachusetts 02139; ‡‡‡ University of Geneva Medical School, 1211 Geneva, Switzerland

**Keywords:** comparative, assembly, vector, malaria, collaboration

## Abstract

We report the imminent completion of a set of reference genome assemblies for 16 species of *Anopheles* mosquitoes. In addition to providing a generally useful resource for comparative genomic analyses, these genome sequences will greatly facilitate exploration of the capacity exhibited by some Anopheline mosquito species to serve as vectors for malaria parasites. A community analysis project will commence soon to perform a thorough comparative genomic investigation of these newly sequenced genomes. Completion of this project via the use of short next-generation sequence reads required innovation in both the bioinformatic and laboratory realms, and the resulting knowledge gained could prove useful for genome sequencing projects targeting other unconventional genomes.

Although the geographic extent of endemic malaria transmission has been curtailed during the last century—in part through intensive mosquito control programs—the World Health Organization estimates that approximately 660,000 deaths were attributable to malaria in 2010 (World Health Organization 2013). The importance of mosquitoes to malaria transmission and control was first established by Ronald Ross more than a century ago, a discovery for which he was awarded the second Nobel Prize in Physiology and Medicine in 1902. Subsequent studies by Batista Grassi and others revealed that only *Anopheles* mosquitoes, and not mosquitoes of other genera such as *Culex* or *Aedes*, were capable of transmitting the disease to humans. There are hundreds of species of *Anopheles* mosquitoes, and a century of medical entomology has established that only a few dozen species are important vectors of human malaria (Budiansky 2002). Even within those species, not all individual mosquitoes or populations are equally competent as vectors. Why is this trait so variable?

The biological basis for variable vectorial capacity surely lies in poorly understood differences in mosquito physiology, molecular biology, and/or behavior. A better understanding of vectorial capacity may ultimately enable its manipulation for the reduction of disease burden. The publication of the African vector *Anopheles gambiae sensu stricto* genome in 2002 (Holt *et al.* 2002) was a landmark for the field of malaria vector research, but gaining a better understanding of vectorial capacity clearly requires a comparative framework. Toward that end, we are sequencing the genomes and transcriptomes of an additional 16 *Anopheles* species. Several of these species belong to the *An. gambiae* sibling species complex (*An. gambiae sensu lato*) and thus are extremely similar phenotypically and genetically, whereas the remaining are more evolutionarily divergent. In addition, we aim to generate genetic polymorphism data for many of these species by sequencing several individuals sampled from natural populations or colonies. Together with the *An. gambiae* s.s. genome, these 17 annotated genome assemblies will provide a platform for gaining genome-wide evolutionary and population genetic insights into the mechanisms of speciation, and the biological processes that influence the ability of mosquitoes to transmit malaria parasites to humans. These processes include detection of host odors, immune responses, and insecticide resistance.

The effort to shape a comparative genomic project was led by Nora Besansky (University of Notre Dame), who, in consultation with other members of the *Anopheles* research community on the *Anopheles* Genomes Cluster Committee, identified 13 mosquito species that captured evolutionary and phenotypic divergence among *Anopheles* mosquitoes and for which quality sequencing template could be generated. The sequencing of two incipient species of *An. gambiae* (“M” and “S” forms) using Sanger technology (Lawniczak *et al.* 2010) originally was executed as a proof-of-principle demonstration immediately before it became clear that next-generation sequencing (NGS) technology would be more appropriate for this larger project. The initial whitepaper was approved in September 2008 by the National Human Genome Research Institute and the National Institute of Allergy and Infectious Diseases of the U.S. National Institutes of Health, after review by the Eukaryotic Pathogens and Disease Vectors Target Selection Working Group. A contract to execute the project was awarded to the Broad Institute. Three additional species were later added to the project when DNA and RNA template sources became available, and a full list of the 16 targeted species and their putative evolutionary relationships are depicted in Figure 1.

**Figure 1:**
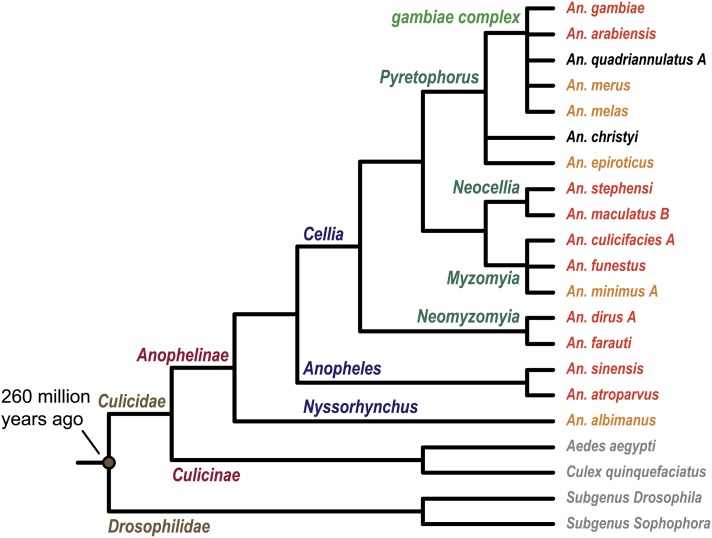
Illustration of the 16 anophelines and their relationships to *An. gambiae*, the two sequenced culicines—*Aedes aegypti* and *Culex quinquefasciatus*—and the sequenced *Drosophila* species. The divergence time estimate between Drosophilidae and Culicidae is from (Gaunt and Miles 2002). Anopheles species that are major human malaria vectors are labeled in red, minor vectors are labeled in orange, and species that are not human malaria vectors are labeled in black.

Many of the community sequencing projects that made first use of the short but economical NGS reads, such as the 1000 [human] Genomes Project (Siva 2008), benefited from the availability of pre-existing high-quality reference assemblies for the target species. Genetic polymorphisms can be readily identified through mapping NGS reads to a reference assembly, in an approach often termed “resequencing.” *De novo* assembly from short NGS reads, however, proved to be a far more difficult prospect. As a result, quality vertebrate NGS-based assemblies did not begin appearing until 2010/2011 (Li *et al.* 2010a,b; Gnerre *et al.* 2011), almost 5 years after the introduction of NGS technology.

The recipe for producing NGS-based vertebrate assemblies, however, proved impractical to transfer directly to mosquitoes. Initial attempts to assemble *Anopheles gambiae s.s* with the ALLPATHS LG algorithm (Gnerre *et al.* 2011) in the same manner as human or mouse were unsuccessful; contig N50 measurements (a weighted median statistic) were on the scale of 2−3 kb, much smaller than necessary to ensure high-quality gene models (Table 1). Clearly, a new approach would be needed to deal with the unique architecture of these mosquito genomes.

**Table 1:** Process improvement in *de novo Anopheles gambiae* Illumina assembly

	Contig N50, kb	Scaffold N50, Mb
Original ALLPATHS LG	2.7	0.049
With haploidify	22	0.349
With haploidify + Fosill reads	26	3.5

*Anopheles* genomes are roughly 10 times smaller than the human genome, at approximately 275 Mb. Nevertheless, they contain a large number of widely dispersed repetitive sequences—in the form of transposon insertions as well as intercalated heterochromatic repeats—that foil attempts at assembly with reads from sequencing libraries made of small DNA fragments. We have found natural populations as well as colonies of most *Anopheles* species to be highly polymorphic, with individuals exhibiting heterozygous base positions at rates up to 10—15 times greater than found in most vertebrates. To address these issues, three strategies have been used in the *Anopheles* 16 Genomes project.

First, old-fashioned mosquito husbandry was used to remove as much genetic diversity as possible from laboratory colonies before preparation of sequencing template. *Anopheles* mosquitoes can be difficult breeders in captivity, but Paul Howell and Alice Sutcliffe at the National Institute of Allergy and Infectious Diseases−funded Malaria Research and Reference Reagent Resource Center (MR4; http://www.mr4.org/) were able to generate subcolonies from single-pair matings for nine of the 12 of the species in this project maintained as captive colonies by MR4. By founding subcolonies with the progeny of single inseminated females (Benedict and Rafferty 2002), Howell and Sutcliffe were able to substantially reduce the polymorphism in tissue used for genomic DNA template relative to the original colonies. To further reduce polymorphism in the template, the small-insert sequencing libraries used in assembly were generated from the DNA of single female mosquitoes rather than pools of individuals, using whole genome amplification to increase the quantity of available DNA required for the 3- to 5-kb insert libraries.

Second, the Broad’s ALLPATHS LG assembly algorithm was modified to deal with the very high heterozygosity rates. Using an approach called “Haploidify” created by Filipe Ribeiro, Iain MacCallum, and others in David Jaffe’s Computational Research and Development team at the Broad, base-calling errors in the data could be better differentiated from legitimate heterozygous positions, and the legitimately heterozygous positions could be “side-stepped” during the assembly process before being restored at the end. This resulted in a 10-fold increase in contig size (Table 1).

Third, pairs of Illumina reads from Fosmid-scale Illumina (“Fosill”) libraries (Williams *et al.* 2012) were found to be critical for achieving good scaffold sizes (Table 1). With read-pairs separated by 38−40 kb, contigs could be efficiently linked up across repetitive islands and heterochromatic regions, and scaffold N50 increased 10-fold. However, the Broad’s original protocol for Fosill libraries had a steep input requirement: 20−25 μg of high-molecular-weight DNA. Generating such template required as many as 500 or more female mosquitoes, an onerous burden for Howell and other template providers. Fortunately, Louise Williams and her colleagues in Andi Gnirke’s Molecular Biology Research and Development team at the Broad were able to modify the insert size-selection component of the Fosill protocol. Using lambda phage packaging to accomplish size selection instead of the original gel-based method, Williams was able to reduce the input requirement to as little as 3 μg, a quantity that was much easier to procure for these organisms. Comparison of the size range of inserts observed in traditional and “gel-free” Fosill libraries indicates highly similar performance (mean insert size = 38.0 and 36.7 kb, respectively; Figure 2).

**Figure 2:**
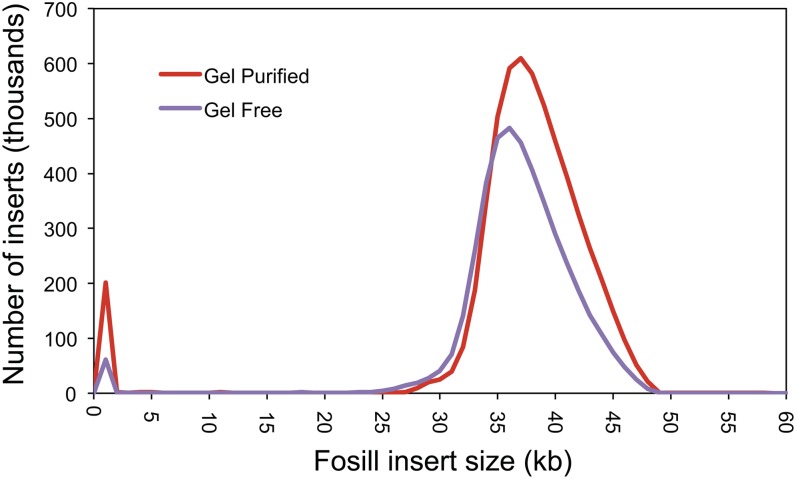
A plot comparing the density of insert sizes observed in two Fosill library preparations: gel-purified and gel-free. The comparable performance of the gel-free library made with less than one fifth of the DNA required for the gel-purified approach (20−25 μg) is an enabling breakthrough for sequencing projects with limited template availability.

After years of development and innovation, both informatic and laboratory-based, and with the contribution of quality sequencing template from many members of the research community, the Broad will be releasing *de novo* genome assemblies for all 16 of the *Anopheles* species in this project during 2013. Draft assemblies will be made available to the community via the Broad website (https://olive.broadinstitute.org/projects/anopheles), VectorBase (https://www.vectorbase.org), and NCBI. The VectorBase team will produce initial gene predictions (Dan Lawson, European Bioinformatics Institute), bringing together *ab initio* and similarity-based approaches informed by protein and transcriptome data. VectorBase, an actively managed repository of vector-related genomic data, will be the long-term home of the *Anopheles* genomes. Assembly improvement using whole-genome alignments and transcriptome-based corrections will be undertaken by VectorBase and Robert Waterhouse (Kellis Laboratory, MIT). Multiple whole-genome alignments and orthology-based approaches to improve initial gene predictions, pioneered by Kellis *et al.* in the *Drosophila* 12 Genomes project (Stark *et al.* 2007), will be performed and integrated with community annotations leading to a final “freeze” of the assemblies and gene predictions by the end of 2013.

Once assemblies and gene predictions are finalized, the community analysis will begin in earnest. Members of the vector community and wider genomics research community interested in the comparative analyses are invited to contact the project organizers. Major analysis themes will include speciation, molecular evolution, chemoreception, circadian rhythm, development, immunity, insecticide resistance, metabolism, repetitive elements, reproduction, the sialome, inversions and chromosomal architectures, neuropeptides, blood/sugar digestion, and transcriptional regulation.

The success of these analyses will depend in part on the accuracy and completeness of the gene predictions for each species. The quality of the gene predictions will in turn depend largely on the contiguity and completeness of the *de novo* assemblies. For some applications, *de novo* assemblies may not be necessary if a closely related taxon has already been sequenced. However, the lack of species-specific assemblies can potentially bias comparative analyses against discovery of those genome regions that are most dynamic and interesting. Although there is appreciation in the research community of the significant value of producing quality *de novo* assemblies for previously unsequenced species, there is less awareness that producing such assemblies remains a technically difficult task, or that the research and development costs associated with assembling genomes with a novel architecture can nullify the low cost of raw NGS data.

In our experience, a readily available DNA sample and cheap sequencing are necessary but insufficient ingredients for the success of sequencing projects that aim to produce *de novo* assemblies. A source of high-quality template must be provided, sometimes in large quantities, and potentially manipulated to reduce heterozygosity. Therefore, the support of the research community is crucial. Innovation in sequencing and assembly approaches will be necessary, drawing from expertise largely found in sequencing centers. Finally, experienced bioinformatics support, such as that provided by a Bioinformatics Resource Center like VectorBase and members of the research community with genomics expertise, is essential for annotation, data curation and efficient resource sharing with the broader community. The *Anopheles* project has been fortunate to leverage all of these resources, allowing it to produce quality reference assemblies for important vectors of malaria and fully capitalize on the research potential of inexpensive sequencing data.
